# Quality of caesarean delivery services and documentation in first-line referral facilities in Afghanistan: a chart review

**DOI:** 10.1186/1471-2393-12-14

**Published:** 2012-03-15

**Authors:** Young-Mi Kim, Hannah Tappis, Partamin Zainullah, Nasrat Ansari, Cherrie Evans, Linda Bartlett, Nabila Zaka, Willibald Zeck

**Affiliations:** 1 Jhpiego, Baltimore, Maryland, USA; 2 Department of International Health, Johns Hopkins Bloomberg School of Public Health, Baltimore, Maryland, USA; 3 Jhpiego, Kabul, Afghanistan; 4 United Nations Children's Fund (UNICEF), Kabul, Afghanistan; 5 Regional Office for South Asia, United Nations Children's Fund (UNICEF), Kathmandu, Nepal

## Abstract

**Background:**

Increasing appropriate use and documentation of caesarean section (CS) has the potential to decrease maternal and perinatal mortality in settings with low CS rates. We analyzed data collected as part of a comprehensive needs assessment of emergency obstetric and newborn care (EmONC) facilities in Afghanistan to gain a greater understanding of the clinical indications, timeliness, and outcomes of CS deliveries.

**Methods:**

Records were reviewed at 78 government health facilities expected to function as EmONC providers that were located in secure areas of the country. Information was collected on the three most recent CS deliveries in the preceding 12 months at facilities with at least one CS delivery in the preceding three months. After excluding 16 facilities with no recent CS deliveries, the sample includes 173 CS deliveries at 62 facilities.

**Results:**

No CS deliveries were performed in the previous three months at 21% of facilities surveyed; all of these were lower-level facilities. Most CS deliveries (88%) were classified as emergencies, and only 12% were referrals from another facility. General anesthesia was used in 62% of cases, and spinal or epidural anesthesia in 34%. Only 28% of cases were managed with a partograph. Surgery began less than one hour after the decision for a CS delivery in just 30% of emergency cases. Among the 173 cases, 27 maternal deaths, 28 stillbirths, and 3 early neonatal deaths were documented. In cases of maternal and fetal death, the most common indications for CS delivery were placenta praevia or abruption and malpresentation. In 62% of maternal deaths, the fetus was stillborn or died shortly after birth. In 48% of stillbirths, the fetus had a normal heart rate at the last check. Information on partograph use was missing in 38% of cases, information on parity missing in 23% of cases and indications for cesareans missing in 9%.

**Conclusions:**

Timely referral within and to EmONC facilities would decrease the proportion of CS deliveries that develop to emergency status. While the substantial mortality associated with CS in Afghanistan may be partly due to women coming late for obstetric care, efforts to increase the availability and utilization of CS must also focus on improving the quality of care to reduce mortality. Key goals should be encouraging use of partographs and improving decision-making and documentation around CS deliveries.

## Background

Caesarean section (CS) is a surgical intervention to prevent or treat life-threatening maternal or perinatal complications. It is widely recognized as an effective intervention to reduce maternal and perinatal mortality when used appropriately [1-4]. Maternal and perinatal deaths associated with labor complications such as malpresentation, obstructed labor, and suspected uterine rupture are generally preventable with timely caesarean section [5]. Appropriate and judicious decision making is vital, however, because a CS delivery that is not medically justified increases the risk of maternal and perinatal mortality compared with an uncomplicated vaginal delivery [6-9]. CS deliveries also expose women to an increased risk of obstetric complications such as uterine rupture [10,11], placenta praevia, and abruption during subsequent pregnancies [12-14]. Increases in national CS rates beyond 15% have been correlated with higher maternal and perinatal mortality [4].

In Afghanistan, improving access to quality emergency obstetric and newborn care (EmONC) services, including caesarean section, has been a priority of the Ministry of Public Health (MoPH) since 2002 [15]. A 2009 National EmONC Needs Assessment in Afghanistan estimated that CS deliveries made up only 1.1% of all births [16]. This low CS rate very likely contributes substantially to the high risk of maternal and newborn mortality [17-19]. The risk is exacerbated among poor and rural women who live in areas without resources for caesarean section and also by indecision and delays in performing a CS delivery. When Afghan women do receive a CS delivery, inconsistent access to EmONC places them at increased risk of complications during subsequent pregnancies.

There have been few studies of CS service delivery and its outcomes in Afghanistan. Two studies reviewed 2006 logbook data from hospitals in the capital city of Kabul. One assessed risk factors for perinatal mortality, including CS delivery [20], while the other measured fetal and maternal deaths related to CS delivery and determined contributing factors [21]. There is a gap in the literature with regard to evaluating the quality of care and appropriate use of caesarean sections across different facility types and regions in Afghanistan. The study reported here is designed to narrow that gap and inform CS scale up by providing an assessment of the quality of CS provision and documentation at EmONC facilities across the country.

## Methods

This study is a cross-sectional, descriptive assessment of 173 CS deliveries in 62 first-line EmONC referral sites based on a record review. It is one component of the 2009 National EmONC Needs Assessment conducted by Jhpiego, an affiliate of Johns Hopkins University, and the MoPH, with financial support from the United Nations Children's Fund (UNICEF).

At the time of the assessment, 127 government health facilities across Afghanistan were designated as comprehensive EmONC (CEmONC) providers. That is, they were supposed to provide nine essential services designated as signal functions for emergency obstetric care by the Averting Maternal Death and Disability (AMDD) Program, the United Nations Population Fund (UNFPA), United Nations Children's Fund (UNICEF), and World Health Organization (WHO). The seven basic signal functions are: parenteral antibiotics, uterotonics, parenteral anticonvulsants, manual removal of the placenta, removal of retained products, assisted vaginal delivery, and neonatal resuscitation. To be designated as a comprehensive service provider, two additional signal functions are required: blood transfusions and caesarean sections [22,23].^a^

**Figure 1:**
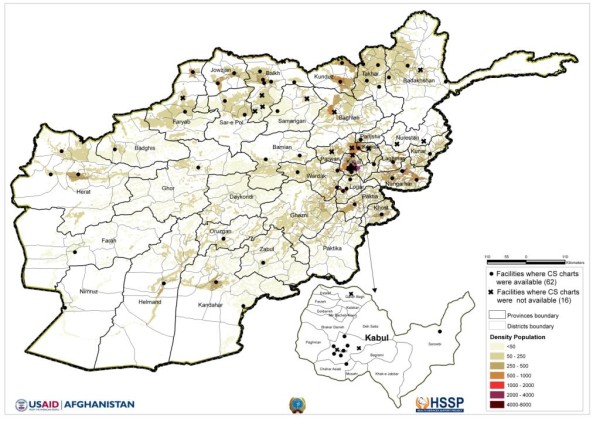
**Geographical distribution of 78 study facilities by availability of CS charts for review**.

Facilities designated as CEmONC providers include district, provincial, regional, and specialized maternity hospitals, as well as certain comprehensive health centers (CHCs). These 'CHC *plus*' facilities are assigned to provide the functions of a district hospital in areas where there is no district hospital. The study plan provided for the assessment of all 127 public CEmONC facilities, but 49 of them were not accessible due to security constraints at the time of the field work. Therefore, the National EmONC Needs Assessment was limited to 78 facilities located in secure areas of the country. This census of secure facilities is representative of 31 of Afghanistan's 34 provinces (91%). (Figure 1 shows their location.) The facilities included 9 CHC *plus *facilities, 34 district hospitals, 25 provincial hospitals, 5 regional hospitals, and 5 specialized maternity hospitals. Two-thirds (67%) were located in urban areas.

For the chart review, we adapted a research tool (module 8 on CS delivery) from the standardized AMDD Program Needs Assessment Toolkit [22]. The tool is designed to assess record keeping and identify indications for cesarean, maternal and newborn outcomes, and various aspects of the quality of care for each case such as the flow of time during patient care and operative and post-operative care practices. Six Afghan doctors and 38 Afghan midwives with experience in obstetric care were recruited to collect the data. They attended a one-week training on all modules of the AMDD Toolkit, including use of the CS chart review research tool and its methodology, and their competence was evaluated during a pilot of the tool at health facilities in Kabul. The data collectors were instructed to obtain consent from each facility's medical director and hold an introductory meeting with other key informants before reviewing patient medical records, facility logbooks, and registers for the three most recent CS deliveries performed in the preceding 12 months. Because CEmONC facilities are expected to provide the same standard of care regardless of the level of facility or frequency of CS deliveries, the same number of cases was drawn from each facility. Facilities were not notified in advance of the visit, and data collectors were external to the facilities they assessed.

The study was approved by institutional review boards at the Afghanistan Public Health Institute and the Johns Hopkins Bloomberg School of Public Health, and consent was obtained from the Medical Director or Officer-in-Charge at each facility.

## Results

### Characteristics and caseloads of the facilities

Sixteen of the 78 facilities surveyed (21%) were excluded from the study because no CS deliveries had been performed in the previous three months. They included 8 of the 9 CHC *plus *facilities (89%) and 8 of the 34 district hospitals (24%). Of these 16 facilities, 3 CHC *plus *facilities reported no cases requiring a CS delivery during this time period. The other 13 facilities reported treating women who needed a caesarean section but did not offer the procedure due to human resource limitations (77%), management issues (54%), lack of supplies or equipment (31%), and training issues (15%) [16].

Despite the exclusion of some facilities, the remaining 62 facilities (which reported at least one CS delivery in the past three months) represent all 31 provinces in the sample, as shown in Figure 1. They include 1 CHC *plus *facility, 26 district hospitals, 25 provincial hospitals, 5 regional hospitals, and 5 specialized maternity hospitals. Of these 62 facilities, 54 facilities (69%) provided three CS cases for review, 4 facilities (5%) provided two cases, and 4 facilities (5%) provided one case, for a total of 173 CS deliveries. Almost half of these cases (46%) came from district hospitals, while 24% came from provincial hospitals and 28% came from regional and specialized hospitals.

The CS caseload varied greatly across the facilities assessed, from one case in the previous 12 months at 1 CHC *plus *facility and 3 district hospitals to 3,105 cases at the largest specialized maternity hospital in Kabul. The three charts reviewed at each facility accounted for an average of 28% (range 1.6%-100%) of the CS cases performed at each district-level EmONC facility in 2009, an average of 11% (range 0.5%-60%) of CS cases at each provincial hospital and less than 2% (range 0.1%-7.9%) of the CS deliveries performed at each national or specialty hospital.

### Characteristics of the women

Women delivering by caesarean were primarily between ages 20 and 29 (37%) and ages 30 and 39 (46%). Only 2% were younger than age 20, and 10% were age 40 or older. Age was not recorded in 10% of cases. Their average age was 28, with a range from 18 to 45.

All of the women enrolled in this study were classified as multiparas: 14% had one child, 38% had two to five children, and 25% had six or more children. Parity was not recorded in 23% of cases. Average recorded parity was 4.2, with a range of 1 to 12 children.

Over two-thirds (68%) of the women resided in rural areas, and 27% lived in urban areas. There was no information on residence for the remaining 5% of women. Only 12% of the 173 women were referred from another health facility.

### Type of caesarean, indications, and partograph use

Overall, most of the CS deliveries (88%, n = 151) were classified as emergencies, that is, they were performed in response to urgent medical complications. Another 10% (n = 18) were classified as non-emergency cases. There was no information for the remaining 2% (n = 4). At provincial hospitals, 17% of the CS deliveries reviewed were non-emergency cases. Non-emergency cases accounted for a smaller proportion of CS deliveries at district, regional, and specialized hospitals (8-9%).

Maternal indications accounted for 61% of CS deliveries, fetal indications for 30%, and there was no information for 9% (Table 1). For emergency caesareans, the most common indications were cephalopelvic disproportion (CPD)/prolonged labor (28%), placenta praevia or abruption (19%), malpresentation (15%), and fetal distress (13%). For non-emergency caesareans, the most common indications were previous scar (22%), CPD/prolonged labor (22%), and malpresentation (22%).

**Table 1:** Percent distribution of CS cases by indication, according to emergency classification

Indication	Emergency cases (N = 151)		Non-emergency cases (N = 18)		All CS deliveries (N = 169)	
	N	%	N	%	N	%
**All maternal indications**	**91**	**60.3**	**12**	**66.6**	**103**	**60.9**
Placenta praevia/abruption	29	19.2	1	5.6	30	17.7
Maternal distress	3	2.0	1	5.6	4	2.4
Failed induction	0	0.0	0	0.0	0	0.0
Previous scar	11	7.3	4	22.2	15	8.9
Eclampsia/severe pre-eclampsia	6	4.0	1	5.6	7	4.1
CPD/prolonged labor	42	27.8	4	22.2	46	27.2
Vesico-vaginal fistula	0	0.0	1	5.6	1	0.6
**All fetal indications**	**48**	**31.8**	**5**	**27.8**	**51**	**30.2**
Cord prolapse/presentation	5	3.3	0	0.0	5	3.0
Fetal distress	20	13.2	1	5.6	21	12.4
Malpresentation	22	14.6	4	22.2	24	14.2
Multiple gestation	1	0.7	0	0.0	1	0.6
**No information**	**12**	**7.9**	**1**	**5.6**	**15**	**8.9**

Information on use of the partograph was available for 107 of the 173 cases reviewed. Only 28% of these cases were managed with a partograph. Use of the partograph was more common at higher level facilities: the partograph was used in 32% of cases at provincial hospitals and in 39% of cases at regional and specialty hospitals. By contrast, the partograph was used in just 24% of cases at district hospitals.

### Characteristics of CS deliveries

General surgeons performed 52% of the CS deliveries reviewed, obstetrician/gynecologists performed 36%, and general practitioners or other staff performed the remaining 12%. General surgeons accounted for a greater portion of the caseload at district hospitals and the CHC *plus *facility (61%) than at higher level facilities (43-45%).

General anesthesia was used in 62% of cases, and spinal or epidural anesthesia in 34%. Spinal/epidural anesthesia was used more frequently at provincial hospitals (in 48% of cases) than at district hospitals and the CHC *plus *facility (27%) or at regional and specialized hospitals (33%). No information was available on the type of anesthesia used in 4% of cases.

**Figure 2:**
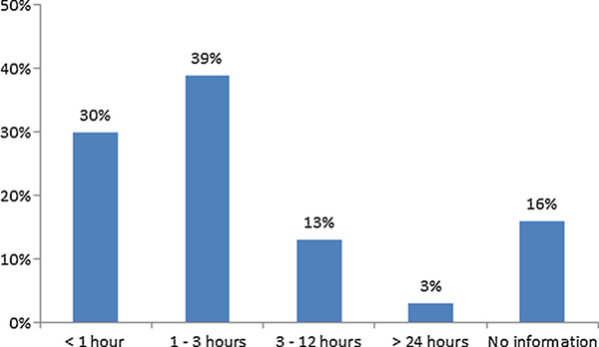
**Delays in performing CS deliveries**. Percent distribution of emergency cesareans by elapsed time between the decision to perform a cesarean and the start of surgery.

When women present with obstetric emergencies, time to treatment matters. In 30% of the 151 emergency cases reviewed, the incision was made less than one hour after the decision to perform a CS delivery (Figure 2). In 38% of emergency cases, surgery began one to three hours after the decision. Delays were even longer in 16% of emergency cases, ranging from 3 hours to more than 24 hours. There was no information on the remaining 16% of emergency cases.

### Maternal outcomes

About three-quarters (74%) of the 173 women survived the CS delivery, 16% died, and there was no information on maternal outcomes in 10% of the cases. Of the 27 cases that resulted in maternal deaths, 24 also reported the death of the fetus and 3 reported twins with mixed outcomes (i.e., one baby survived and one died). Four of the 27 cases that resulted in maternal death were cases classified as 'non-emergencies' and one was missing classification. In 52% of cases of maternal death, the leading indication for the CS delivery was maternal, usually placenta praevia or abruption (41%) (Table 2). In the remaining 41% of maternal deaths, the indication for CS delivery was fetal, with malpresentation (30%) the leading cause. There was no information on indications in the other 7% of cases.

**Table 2:** Percent distribution of CS cases by indication, according to maternal and fetal outcomes

Indication	Live birth (N = 136)		Maternal death (N = 27)		Early neonatal death (N = 3)*		Stillbirth (N = 30)**	
	N	%	N	%	N	%	N	%
**All maternal indications**	**86**	**63.0**	**14**	**51.9**	**0**	**0.0**	**16**	**53.3**
Placenta praevia/abruption	18	13.3	11	40.7	0	0.0	12	40.0
Maternal distress	3	2.2	2	7.4	0	0.0	1	3.3
Previous scar	14	10.3	0	0.0	0	0.0	0	0.0
Eclampsia/severe pre-eclampsia	7	5.2	0	0.0	0	0.0	0	0.0
CPD/prolonged labor	43	31.9	1	3.7	0	0.0	3	10.0
Vesico-vaginal fistula	1	0.7	0	0.0	0	0.0	0	0.0
**All fetal indications**	**39**	**28.9**	**11**	**40.7**	**3**	**100.0**	**10**	**33.3**
Cord prolapse/presentation	1	0.7	2	7.4	0	0.0	2	6.7
Fetal distress	19	14.1	1	3.7	1	33.3	1	3.3
Malpresentation	18	12.9	8	29.6	2	66.6	7	23.3
Multiple gestation	1	0.7	0	0.0	0	0.0	0	0.0
**No information**	**11**	**8.1**	**2**	**7.4**	**0**	**0.0**	**4**	**13.3**

Fetal outcomes were unknown in 5 CS cases

*Twins with mixed outcomes accounted for one early neonatal death; the indication was fetal distress

**Twins with mixed outcomes accounted for two stillbirths; the indication for both was malpresentation

Records show that the partograph was used in 41% of cases of maternal death; it was not used in 52% of these cases; and the information was missing for 7% of cases. The proportion of CS deliveries that ended in maternal death was similar for general surgeons (18%) and obstetrician/gynecologists (15%).

More than one-third (35%) of women who survived the CS delivery (43 of 129 cases) received tubal ligations for family planning during the surgery. All of these women gave verbal or written informed consent for tubal ligation.

### Fetal outcomes

Most (78%) of the 173 CS deliveries reviewed resulted in a live birth. The fetus died during or shortly after delivery in 30 cases (17%), and outcomes were mixed for 3 cases involving twins (2%). Of these 33 perinatal deaths, 30 were fetal deaths and 3 were early neonatal deaths. The mother also died in 27 of these 33 cases (87%).

In 53% of stillbirths, the leading indication for caesarean surgery was maternal, most often placenta praevia or abruption (40%) (Table 2). In 33% of stillbirths, the indication for CS delivery was fetal, with malpresentation (23%) the leading cause. There was no information on indications in the other 13% of cases. In the three cases of early neonatal death, the indications were malpresentation and fetal distress. Meconium was present in 34% of the CS deliveries reviewed, although 18% of cases were missing this information.

The fetal heart rate was checked and recorded at some point during care in 93% of all cases assessed. The last fetal heart rate recorded prior to surgery was normal in 31% of cases and abnormal in 38%; there was no detectable heartbeat in 9% of cases. There is no information on the remaining 15% of cases. Among the 27 CS deliveries resulting in a stillbirth with information on the fetal heart rate, 59% had a detectable heartbeat at the last check: 48% had a normal heart rate and 11% had an abnormal fetal heart rate. There was no detectable heartbeat in 41% of these cases. One of the three early neonatal deaths had an abnormal heart rate.

In 53% of the 62 emergency cases that recorded an abnormal fetal heartbeat, surgery began one to three hours after the decision for a CS delivery. No surgeries began sooner. Delays ranged from 3 hours to more than 24 hours in 19% of cases, and there was no information on timing for the remaining 34% of cases. In two of the three non-emergency cases that recorded abnormal fetal heartbeats, surgery began three to five hours after the decision for a CS delivery; surgery was delayed 72 hours in the third case. It is not possible to calculate the amount of time that elapsed from the first recorded abnormal heartbeat to surgery.

Records show that the partograph was used in 13% of cases of fetal death; it was not used in 38% of these cases and no information was available for the remaining 49% of cases. The proportion of CS deliveries that ended in fetal death was slightly higher for general surgeons (22%) than obstetrician/gynecologists (16%).

### Chart completeness

When asked about their observations of the completeness and updating of facility records, Medical Directors at 95% of facilities in this study reported that all columns in their labor and delivery ward registers were complete and 97% reported that all columns in their operating theatre logbooks were complete. Only one district hospital and one provincial hospital reported incomplete operating theatre logbooks, while three district hospitals reported incomplete labor and delivery ward registers. Although registers and logbooks may have been complete, many charts reviewed were missing data on patient characteristics, indications, and operative or post-operative procedures. Information on partograph use was missing in 38% of cases (n = 66), information on parity missing in 23% of cases (n = 39), indications for cesareans missing in 9% (n = 15), information on anesthesia use missing in 6% of cases (n = 10), fetal outcomes missing in 3% of cases (n = 5), and emergency classification missing in 2% of cases (n = 4).

## Discussion

### Raising CS rates

CS deliveries make up just 1% of all expected births in Afghanistan [16], so the procedure is grossly underused on a national level. This study identified some factors contributing to the low CS rate. Many lower level facilities designated by the MoPH to provide CEmONC services perform few, if any, CS deliveries. Twenty-one percent of the 78 facilities in the study reported no CS deliveries in the previous three months, and an additional 10% of facilities reported only one or two CS deliveries in the past year. This is not surprising given the documented shortages of essential equipment, personnel, and expertise needed for surgical interventions like caesarean sections, and related services such as blood transfusion, especially at lower level facilities [16,24].

Maintaining providers' clinical skills and confidence is challenging in low-volume settings such as these and may require special strategies. For example, regular targeted refresher trainings, clinical audits such as maternal death reviews, and new quality assurance methods (such as drills where a mock emergency is staged and practice and procedures rehearsed) can ensure that providers identify clinical indications of obstetric complications and address them in a timely fashion. Conducting CEmONC simulation trainings for staff based at lower level facilities may serve a dual purpose: it may motivate female providers to work in these relatively remote facilities as well as improve their skills. Another way to keep CS providers' skills sharp is to rotate them from low volume facilities to busier facilities. The alternative-- reclassifying CEmONC facilities with limited caseloads so that they refer women who need a CS delivery to other facilities--is not a good option. The existing CEmONC facilities are a bare minimum with coverage of 1.06 facility per 500,000 population, and Afghanistan's mountainous terrain makes transportation difficult and referral a practical impossibility for many women. Therefore, the priority should be to continue to expand CS coverage by building the surgical capacity of lower level CEmONC facilities that serve the rural areas where most Afghans live. Strengthening CS capacity at lower-level facilities will also increase equity in the provision of CS deliveries.

Poor decision-making by providers also contributes to the low CS rate. The limited number of referrals in this study (just 12% of all cases) suggests that providers at lower-level facilities do not always identify women in need of CS deliveries and refer them to a CEmONC facility. Once women arrive at a CEmONC facility, decisions may not always be made appropriately. Although this study does not shed much light on the decision-making process at CEmONC facilities, missing data on indications for CS in patient charts at both higher and lower-level facilities, and reports in Kabul of CS deliveries for inappropriate indications, such as premature rupture of the membranes, suggest that Afghan providers do not always follow evidence-based recommendations for CS deliveries [21]. Further investigation is needed, but steps must be taken to encourage appropriate referrals for the procedure and to improve the quality of decision-making around CS deliveries at CEmONC facilities. One way to encourage facilities to refer difficult cases may be to require receiving facilities to report back to referring facilities about the arrival and outcome of these cases.

Rising CS rates worldwide, including in developing countries, have raised concern about unnecessary procedures wasting scarce health care resources and driving up maternal and neonatal mortality rates [25]. This study found no evidence for psychosocial indications-- that is, conducting a CS delivery at the mother's request without any medical justification-- which has increased CS rates in other countries [26]. The study was limited to the public sector, however, and did not include private facilities patronized by wealthier women who might choose elective CS deliveries for reasons of convenience or reluctance to have a vaginal delivery. Although unjustified CS deliveries do not currently pose much of a problem in Afghanistan, any efforts to increase CS rates should emphasize the quality of good decision-making and the importance of decreasing unnecessary procedures.

### Improving the quality of care

The findings point to multiple deficits in the quality of care, which no doubt contributed to the large number of maternal and fetal deaths in this sample of CS deliveries. The vast majority (87%) of CS deliveries were performed on an emergency basis, which carries greater risks for maternal complications than planned CS deliveries [27-29]. Quality care, including the timely diagnosis of complications, can prevent medically necessary CS deliveries from becoming emergency cases. For example, clinical signs can alert health personnel to the possibility of placenta praevia--which was a leading cause of death in this study--in mid- to late pregnancy. If the condition is confirmed on ultrasonography, a CS delivery can be arranged [30]. (Ultrasound facilities are widely available in major private and urban clinics in Afghanistan and are frequently consulted to learn the sex of the baby.) However, placenta praevia and other complications are less likely to be detected in Afghanistan, because of low levels of antenatal care (30% in rural areas and 71% in urban areas) and skilled birth attendance (15% in rural areas and 69% in urban areas) [31]. The supply of and demand for antenatal care and skilled birth attendance, as well as its quality, needs to be strengthened in order to detect conditions that require a CS delivery as early as possible.

Research shows that use of the partograph to manage labor results in better outcomes for mother and baby by increasing timely and appropriate interventions--including CS deliveries--when medically necessary and by avoiding unnecessary CS deliveries when labor is not prolonged [32]. Partographs are not needed if a CS delivery is conducted before labor or if a woman arrives in critical condition, but even so partograph use was low in this study, especially at lower level facilities, and lack of information on partograph use was the most common reason for incomplete charts. Use of the partograph for decision-making during routine labor can be encouraged with further instruction for providers in the importance and use of partographs, along with consistent supervision, feedback, and periodic refresher training. Involving a broad range of stakeholders, including pre-service educators, professional organizations, clinical supervisors, and hospital administrators, can help create the enabling environment needed to implement and sustain the change. Further research to explore the barriers and facilitators to partograph use in Afghanistan would help to guide further interventions to increase use.

Poor quality of care also manifests itself in the lack of inductions and assisted vaginal deliveries, which can decrease the need for CS delivery in some cases. There were no cases in this study with failed induction as an indication for CS delivery. This suggests a lack of equipment, knowledge, and/or confidence on the part of skilled birth attendants, which may lead them to avoid inductions and move too early to perform CS deliveries. The National EmONC Needs Assessment in Afghanistan found low rates of assisted vaginal delivery due, in part, to lack of equipment and weak provider skills in using vacuum extractors and forceps [16]. Together these findings suggest a need for additional training, supervision, and equipment to encourage providers to perform inductions and assisted vaginal deliveries and avoid unnecessary CS deliveries.

Although time is of the essence in obstetric emergencies, only 30% of emergency CS surgeries began less than one hour after the decision for a CS delivery was made, and 16% were delayed more than 3 hours. Conducting a full patient flow analysis can help facilities identify the source of delays between the decision for a CS delivery and actual surgery. Maternal death and near miss audits are recommended to identify when, where, and why delays occur. Reasons for delay may include the reluctance of providers to perform a risky procedure, mostly due to a lack confidence in their skills, but also because of potential legal repercussions if outcomes are poor. Medicolegal awareness for providers and families may help ensure providers' rights in these cases. Other reasons include other patients in operating rooms, limited staff or resource availability, or delays in anesthesia services [33,34]. In Afghanistan, for example, limited staffing poses a challenge at provincial hospitals, district hospitals, and CHC *plus *facilities, where gynecologists and surgeons are on call--rather than onsite--at night and on weekends. In addition, most of these facilities only have one operating theater and a single operating table that is shared by the general surgery and obstetric departments. Once these and other sources of delay in CS deliveries are identified, conducting obstetric case simulations could give staff an opportunity to identify bottlenecks in responding to obstetric complications and improve teamwork.

Finally, fetal outcomes in this sample of CS deliveries suggest that the quality of intrapartal care is also poor. The fetus was stillborn or died shortly after birth in all CS cases that ended in maternal death. In addition, the fetus had a normal heart rate at the last check prior to surgery in 48% of deliveries resulting in stillbirth. It is possible that some early neonatal deaths were recoded as stillbirths out of fear of prosecution or families' reactions. Nonetheless, improving newborn care, especially during the first moments of the baby's life, is of utmost importance. Upgrading the skills and attitudes of the operating team is essential, as is strengthening supervision and monitoring systems. Perinatal deaths should trigger facility-based audits that can identify specific practices that need improvement.

### Differences by facility level

It is important to note that the study cases are relatively few in number and do not reflect the actual distribution of CS deliveries by facility type in Afghanistan. Because the same number of cases was drawn from each facility, regardless of caseload, lower level facilities are over-represented and higher level facilities are under-represented. The 78 facilities included in the National EmONC Needs Assessment reported a total of 10,986 CS deliveries over a one-year period [16]. District hospitals and CHC *plus *facilities accounted for less than 6% of these deliveries, but they contributed 47% of the cases reviewed in this study. Specialized and regional hospitals accounted for 82% of all CS deliveries in Afghanistan, but they contributed only 28% of the cases reviewed in this study.

Nonetheless, the findings reveal some key differences in the services offered at various types of facilities. Non-emergency cases made up a greater proportion of CS deliveries at provincial hospitals (17%) than at other facilities (8%-9%). This may reflect staffing patterns for doctors and anesthetists. At remote, rural CHC *plus *facilities and district hospitals, the position of gynecologist often goes unfilled, so general surgeons handle more of the CS caseload. However, general surgeons tend to do emergency life saving procedures only. At provincial hospitals, the one gynecologist on staff is always on call; she may try to schedule CS deliveries in order to avoid emergencies at night or on the weekend. At regional and specialty hospitals, teams of gynecologists work around the clock so there is less pressure to schedule non-emergency CS deliveries.

Limited training and a lack of supplies have slowed the adoption of regional anesthesia in many developing countries [35]. Lack of capacity probably accounts for the limited use of spinal/epidural anesthesia at district hospitals and CHC *plus *facilities, compared with provincial hospitals. However, it does not explain the underuse of regional anesthesia at regional and specialty hospitals. It is possible that staff at these hospitals, who must cope with heavy caseloads, opt for general anesthesia because it is quicker to provide. Lack of awareness that CS deliveries can be performed with regional anesthesia and lack of adequate equipment and knowledge of how to use it may also contribute to its underuse.

### Methodological issues

The study sample is not nationally representative and may overstate the performance of the public health system. Security concerns limited the study to 78 out of 127 EmONC facilities. Those facilities are likely to perform better than the ones excluded from the study. For example, facilities in non-secure areas suffer from a lack of female service providers, especially gynecologists, according to data from Afghanistan's Health Management Information System (HMIS). The study sample was further biased by the dropping of 16 facilities in secure areas that did not report a CS delivery in the preceding three months. Because of their lack of experience with CS deliveries, these facilities are likely to underperform EmONC services compared to the facilities remaining in the sample.

The findings also raise some concern that adolescents and first births are under-represented in the sample of CS deliveries. Although early marriage and adolescent childbearing are common in Afghanistan [31], only 2% of the 173 women in this study were less than 20 years old and none was delivering their first child according to medical records. Both statistics are questionable. Age heaping is common in Afghanistan so many teenagers may have been incorrectly classified as age 20. The number of children recorded likely included the current CS birth, so that some of the women recorded as parity one were probably misclassified and were instead nulliparas delivering their first child. However, with Afghanistan's low rate of institutional delivery (only 14% of births in Afghanistan in 2006 were institutional deliveries), it is possible that there was no misclassification of parity in the charts and that Afghan women are less likely to seek care for their first delivery [36].

The study sample differs from national statistics in other ways. Even if the medical records overstate women's parity by one child, the average parity of women in the study (4.2 children) is still less than the national total fertility rate (TFR) of 6.2 children per woman [31]. This might be due to the under-reporting of female infants. Also, 35% of the women had ever used a contraceptive, which is about 10 percentage points higher than the national contraceptive prevalence rate [31]. It is possible that women who have access to EmONC services are more urban, less poor, more educated and may be more likely to have access to contraceptive services and have lower parity than women in other areas of the country.

Various methods have been used to collect data on the quality of clinical services, including direct observation of services, interviews with health care providers, and the chart review employed by this study. There is little evidence regarding the relative effectiveness of these methods for assessing the quality of clinical services in developing countries. However, the literature tends to support the use of multiple, complementary data collection methods to capture different aspects of the management of labor, delivery, and postpartum care [37,38]. This has been the approach taken by national EmONC assessments in Angola, Ethiopia, and Sierra Leone, all of which have conducted chart reviews of CS deliveries using the AMDD tool as part of a comprehensive assessment of infrastructure, supplies, drugs, and human resources as well as service quality [39-41]. In Afghanistan, the CS delivery chart review tool was also used in the context of a comprehensive national EmONC assessment that employed multiple data collection methods [16].

Chart review offers several advantages over other assessment methods. Compared with observations of clinical care, chart review is less expensive and less intrusive. It also can collect information on maternal and newborn outcomes that occur after surgery, such as infections and early neonatal deaths, although it cannot identify long-term morbidities resulting from CS delivery. Compared with provider interviews, chart review does not impinge on providers' time and may be more objective, since providers may report performing a skill because they are supposed to do so, not because they actually performed the skill. Another advantage of the chart review is that it allows researchers to identify facilities with poor quality reporting and documentation that could benefit from increased attention to completeness and accuracy of medical registers and patient charts.

As with any research method, chart review also has limitations. First, no validity study has been conducted of the AMDD chart review tool, although it is based on best practices and has been deployed in several developing countries. Second, chart reviews are only as reliable a source of information as the quality of record keeping in health facilities. The chart review process accepts the information in patient records at face value, but the accuracy of the data may vary between facilities, or between providers within facilities, and can only be verified through comparison of case records with direct observations.

## Conclusions

Low CS rates undoubtedly contribute to high maternal and perinatal mortality in Afghanistan. However, CS deliveries also carry substantial risks for women and babies due to poor decision making and poor quality of care at EmONC and referral facilities. Thus, efforts to increase the proportion of CS deliveries must also work to improve the quality of care, especially at lower level facilities that extend EmONC services to women living in remote rural areas. Key goals should be improving decision-making around CS deliveries, reducing delays in emergency cases, encouraging the use of partographs, and decreasing the proportion of CS deliveries performed on an emergency basis. This will require additional training, supervision, and feedback for providers at referral as well as EmONC facilities, along with essential equipment and supplies, to ensure that they follow evidence-based recommendations for CS deliveries and use good technique. Monitoring and evaluation activities, such as patient flow analysis and maternal death audits, can help identify problems and potential solutions. Involving a broad range of stakeholders will help create an enabling environment to sustain the changes.

## Endnote

^a^AMDD, UNFPA, UNICEF and WHO use the term 'emergency obstetric care (EmOC)' when referring to the signal functions, the facilities that provide all signal functions. However, for the purposes of this study, the term 'emergency obstetric and newborn care (EmONC)' was used to classify facilities to reflect the inclusion of indicators related to procedures to address neonatal complications during labor and delivery.

## Competing interests

The authors declare that they have no competing interests. This study was one component of a national emergency obstetric and newborn care needs assessment funded by UNICEF and conducted by Jhpiego, an affiliate of the Johns Hopkins University, in collaboration with the Ministry of Public Health of Afghanistan. Any opinions stated are those of the authors and not of UNICEF, Jhpiego, or the Johns Hopkins Bloomberg School of Public Health.

## Authors' contributions

YMK designed the study, served as the Primary Investigator, and coordinated the manuscript drafting and finalization process. HT conducted the descriptive data analysis and wrote major sections of the background, results, discussion, and conclusion sections. She also contributed to critical review and revision of the manuscript. P and NA participated in the design and implementation of the study, contributed to data analysis and critical review of the manuscript, and provided key input into the analysis of results, discussion, and conclusions. CE, LB, NZ, and WZ wrote parts of the manuscript and provided key input into the analysis approach, discussion, and conclusions. All authors read and approved the final manuscript.

## Authors' information

YMK (EdD, MS) is a Senior Monitoring & Evaluation Advisor at Jhpiego's Baltimore office. HT (MPH) is a Researcher and DrPH Candidate at the Johns Hopkins Bloomberg School of Public Health. P (MD, MSc) is a Monitoring and Evaluation Manager at Jhpiego's Afghanistan Office. NA (MD) was the Technical Director of the Health System Strengthening Program at Jhpiego's Afghanistan office; he is currently pursuing a master's degree in Amsterdam. CE (DrPH, CNM) is a Senior Maternal and Newborn Health Technical Advisor at the Jhpiego's Baltimore office. LB (MD, BMedSc, MH) is an Associate Scientist at the Johns Hopkins Bloomberg School of Public Health. NZ (MD, PhD) is a Health Specialist with UNICEF/Afghanistan. WZ (MD, MA) is a Regional Mother & Newborn Health Specialist with the UNICEF Regional Office for South Asia (ROSA), Nepal.

## Pre-publication history

The pre-publication history for this paper can be accessed here:

http://www.biomedcentral.com/1471-2393/12/14/prepub
